# Association between life-style, metabolic syndrome and lower urinary tract symptoms and its impact on quality of life in men ≥ 40 years

**DOI:** 10.1038/s41598-022-10904-7

**Published:** 2022-04-27

**Authors:** Ji Bong Jeong, Jung Hoon Lee, Min Soo Choo, Dong-Won Ahn, Su Hwan Kim, Dong Seok Lee, Min Chul Cho, Hwancheol Son, Hyeon Jeong, Sangjun Yoo

**Affiliations:** 1 Department of Internal Medicine, Seoul National University Boramae Medical Center, Seoul, Republic of Korea; 2 Department of Urology, Seoul National University Boramae Medical Center, Sindaebang 2(i)-dong, Dongjak-gu, Seoul, 07061 Republic of Korea

**Keywords:** Urology, Prostate, Metabolic disorders

## Abstract

We aimed to assess the relationship between lifestyle-related variables, metabolic syndrome, and lower urinary tract symptoms (LUTS) in men ≥ 40 years. We also assessed the impact of these variables on quality of life. From 2014 to 2020, 5355 men who underwent health check-ups with I-PSS questionnaires at our institute were included in the analysis. The impact of LUTS on sleep disorders and moderate to severe degrees of stress were assessed. Multivariate analysis was performed to determine the variables associated with LUTS and prostate volume. Moderate and severe LUTS were present in 1317 (24.6%) and 211 (3.9%) men, respectively. Moderate and severe LUTS were significantly associated with the presence of sleep disorders and stress. On multivariable analysis, age, amount of life-long smoking, marital status, income, job, and decreased HDL-cholesterol were associated with the presence of moderate to severe LUTS. Although older age and the amount of life-long smoking was associated with both voiding and storage sub-score, socioeconomic status, including marital status and income were only associated with storage sub-score. In men ≥ 40 years, stable socioeconomic status, in addition to older age, and life-long smoking amount are associated with the presence of moderate to severe LUTS, which worsens sleep quality and stress level, by worsen storage sub-score.

## Introduction

Recently, as life expectancy has increased, interest in the quality of life (QoL) in elderly individuals is rapidly increasing. In elderly men, lower urinary symptoms (LUTS) were reported to significantly worsen QoL^1,2^. However, currently, no clear strategies to prevent the development of LUTS have been developed, despite more than 40% of men suffering from at least one LUTS^3^. When we consider the medical costs for relieving LUTS in elderly men^4^ and its impact on individual QoL, the development of strategies to prevent LUTS in men is urgently needed.

Maintaining a positive health status is thought to be important for improving QoL, and preventing metabolic syndrome (MetS) is one of the most important concepts for maintaining a positive health status, especially when a person gets older^5–7^. Traditionally, MetS has been regarded as an important risk factor for cardiovascular or cerebrovascular events^5^. However, recently, the relationship between MetS and other chronic diseases has been increasingly reported^6,7^, and LUTS and benign prostatic hyperplasia (BPH) in men have also been reported to be associated with MetS and its components^8^.

In our previous studies, we identified that MetS and its components, especially decreased high-density lipoprotein (HDL) cholesterol, are related to the presence of prostatic disease, including BPH and prostate cancer, using a large national health insurance database cohort^9,10^. However, because the severity of LUTS has not been routinely measured as a health check-up in the normal population, there are only a few studies that address the impact of MetS and its components on the severity of LUTS, and these studies did not show consistent results^11–14^. This might be due to the complex correlation between lifestyle variables, MetS, and LUTS; to elucidate the real impacts of MetS and lifestyle factors on LUTS, thorough analysis using a large database with detailed information is needed.

In our institute, the International Prostate Symptom Score (I-PSS) questionnaires were routinely administered in men who visited the health check-up center. In this study, we aimed to assess the relationship between MetS/lifestyle-related variables and LUTS using these databases, in addition to its impact on QoL. We evaluated the impacts of LUTS on sleep quality and stress to address the effects of improving LUTS on QoL. In addition, we assessed the association between the severity of LUTS and metabolic components and lifestyle-related variables to elucidate the prevention strategies for moderate to severe LUTS to improve QoL by lifestyle modification. We also assessed the impact of these variables on prostate volume to demonstrate the possible reasons for moderate to severe LUTS in these men.

## Results

Among the study population, moderate and severe LUTS were present in 1317 (24.6%) and 211 (3.9%) men, respectively (Table 1). In addition to age, obesity, marital status, job, monthly income, life-long smoking amount, physical activity, and decreased HDL-cholesterol levels were significantly associated with the severity of LUTS. The presence of sleep disorders and moderate to severe stress were also significantly higher as LUTS severity increased. The PSA level and prostate volume were higher in men with severe LUTS.

**Table 1:** Baseline characteristics according to severity of LUTS.

	None to mild	Moderate	Severe	*P*
N (%)	3827 (71.5)	1317 (24.6)	211 (3.9)	
Age, years, mean ± SD	52.4 ± 8.8	56.5 ± 9.4	59.0 ± 9.7	< 0.001
Body weight, kg, mean ± SD	72.4 ± 10.4	70.7 ± 9.9	69.3 ± 10.4	< 0.001
BMI, kg/m^2^, mean ± SD	24.7 ± 2.9	24.4 ± 2.9	24.2 ± 3.1	< 0.001
Waist circumference, cm, mean ± SD	87.1 ± 8.2	87.0 ± 8.1	87.3 ± 8.6	0.858
Marital status, n (%)				< 0.001
Yes	3449 (90.1)	1161 (88.2)	164 (77.7)	
Others	354 (9.3)	140 (10.6)	44 (20.9)	
Unknown	24 (0.6)	16 (1.2)	3 (1.4)	
Job, n (%)				< 0.001
Office-worker	2678 (70.0)	752 (57.1)	90 (42.7)	
Others	1028 (26.9)	504 (38.3)	103 (48.8)	
Unknown	121 (2.3)	61 (4.6)	18 (8.5)	
Monthly income, n (%)				< 0.001
< $2684	3221 (84.2)	985 (74.8)	127 (60.2)	
≥ $2684	455 (11.9)	250 (19.0)	67 (31.8)	
Unknown	151 (3.9)	82 (6.2)	17 (8.1)	
Current smoker, n (%)	1169 (30.5)	380 (28.9)	68 (32.2)	0.414
Amount of smoking, PY, mean ± SD	11.6 ± 13.7	14.8 ± 16.7	19.0 ± 19.5	< 0.001
Drinking, g/week, mean ± SD				0.310
0 g/week	997 (26.1)	354 (26.9)	60 (28.4)	
> 0 and < 100 g/week	875 (22.9)	291 (22.1)	43 (20.4)	
≥ 100 g/week	1876 (49.0)	649 (49.3)	99 (46.9)	
Unknown	79 (2.1)	23 (1.7)	9 (4.3)	
ln (MET), mean ± SD	5.6 ± 3.0	5.6 ± 3.0	3.0 ± 5.0	0.015
Metabolic syndrome, n (%)	1395 (36.5)	516 (39.2)	85 (40.3)	0.137
Central obesity, n (%)	1419 (37.1)	468 (35.5)	80 (37.9)	0.567
Hypertension, n (%)	2355 (61.5)	800 (60.7)	132 (62.6)	0.824
Diabetes, n (%)	1370 (35.8)	502 (38.1)	90 (42.7)	0.058
Triglyceridemia, n (%)	1521 (39.7)	567 (43.1)	86 (40.8)	0.108
Decreased HDL, n (%)	1126 (29.4)	476 (36.1)	69 (32.7)	< 0.001
Sleep disorder, n (%)	1725 (45.4)	802 (61.6)	154 (74.4)	< 0.001
Stress, n (%)	1160 (30.3)	541 (41.1)	115 (54.5)	< 0.001
PSA, mL, mean ± SD	1.6 ± 1.6	1.9 ± 5.1	2.6 ± 4.4	< 0.001
Prostate size, cc, mean ± SD (n = 827)	25.2 ± 7.8	25.5 ± 7.8	28.9 ± 13.1	0.001

Sleep disorders were significantly higher in men with moderate and severe LUTS. Although all components of sleep disorder were significantly related to the severity of LUTS, among components of sleep disorder, sleep disturbance (r = 0.322, *p* < 0.001) was most closely associated with the severity of LUTS, followed by subjective sleep quality (r = 0.252, *p* < 0.001), sleep latency (r = 0.184, *p* < 0.001), and daytime dysfunction (r = 0.161, *p* < 0.001). In multivariate analysis, moderate and severe LUTS were significantly associated with the presence of sleep disorder, in addition to older age, current smoker, increased amount of smoking, marital status, alcohol consumption ≥ 100 g/week, and decreased physical activity (Table 2). Moreover, moderate and severe LUTS were associated with moderate to severe stress, in addition older age, current smoking, increased amount of smoking, marital status, low income, and decreased physical activity.

**Table 2:** Variables associated with quality of life.

	Multivariable	
	OR (95% CI)	P
**(1) Variables associated with the presence of sleep disorder**		
Age, years (continuous)	0.983 (0.976–0.990)	< 0.001
Current smoker (yes vs. no)	1.329 (1.164–1.519)	< 0.001
Amount of smoking, PY (continuous)	1.009 (1.004–1.014)	0.001
Marital status, n (%)		
Yes	Reference	
Others	1.668 (1.372–2.026)	< 0.001
Unknown	0.940 (0.487–1.816)	0.854
Job, n (%)		
Office-worker	Reference	
Others	1.043 (0.913–1.193)	0.534
Unknown	1.560 (1.127–2.160)	0.007
Alcohol consumption, n (%)		
0 g/week	Reference	
> 0 and < 100 g/week	0.965 (0.823–1.132)	0.661
≥ 100 g/week	1.288 (1.123–1.477)	< 0.001
Unknown	0.875 (0.583–1.313)	0.518
ln (MET) (continuous)	0.979 (0.961-.0998)	0.029
IPSS severity, n (%)		
Normal to mild	Reference	
Moderate	2.012 (1.761–2.300)	< 0.001
Severe	3.422 (2.467–4.747)	< 0.001
**(2) Variables associated with the presence of moderate to severe stress**		
Age, years (continuous)	0.957 (0.949–0.964)	< 0.001
Current smoker (yes vs. no)	1.371 (1.192–1.576)	< 0.001
Amount of smoking, PY (continuous)	1.006 (1.002–1.011)	0.005
Marital status, n (%)		
Yes	Reference	
Others	1.670 (1.374–2.030)	< 0.001
Unknown	0.961 (0.477–1.936)	0.911
Income, n (%)		
≥ $2684	Reference	
< $2684	1.281 (1.056–1.555)	0.012
Unknown	0.995 (0.722–1.371)	0.974
ln (MET) (continuous)	0.982 (0.963–1.002)	0.074
IPSS severity, n (%)		
Normal to mild	Reference	
Moderate	1.861 (1.622–2.136)	< 0.001
Severe	3.196 (2.374–4.302)	< 0.001

On multivariable analysis, older age, increased amount of life-long smoking, marital status, low income, non-office workers, and decreased HDL cholesterol levels were significantly associated with the presence of moderate to severe LUTS (Table 3). Older age, marital status, and low income were also significantly associated with the presence of severe LUTS. On multivariable linear regression analysis, older age, increased amount of life-long smoking, and low income was associated with the I-PSS total score (Table 4). Voiding sub-score was associated with older age, increased amount of life-long smoking, and the presence of hypertension. Storage sub-score was associated with older age , increased amount of life-long smoking, marital status, and low income.

**Table 3:** Variables associated with the severity of LUTS.

	Moderate to severe LUTS				Severe LUTS			
	Univariate		Multivariable		Univariate		Multivariable	
	OR (95% CI)	*P*	OR (95% CI)	*P*	OR (95% CI)	*P*	OR (95% CI)	*P*
Age (continuous)	1.054 (1.047–1.061)	< 0.001	1.044 (1.036–1.052)	< 0.001	1.062 (1.04–1.076)	< 0.001	1.050 (1.034–1.066)	< 0.001
Current smoker	0.943 (0.828–1.074)	0.377			1.104 (0.822–1.482)	0.512		
Amount of smoking	1.017 (1.013–1.021)	< 0.001	1.011 (1.007–1.015)	< 0.001	1.023 (1.016–1.030)	< 0.001	1.017 (1.009–1.024)	< 0.001
Marital status, n (%)								
Yes	Reference		Reference		Reference		Reference	
Others	1.353 (1.120–1.635)	0.002	1.248 (1.015–1.535)	0.036	2.504 (1.772–3.537)	< 0.001	2.062 (1.417–3.000)	< 0.001
Unknown	2.061 (1.125–3.774)	0.019	1.391 (0.709–2.726)	0.337	2.108 (0.646–6.885)	0.217	1.287 (0.362–4.570)	0.696
Income								
≥ $2684	Reference		Reference		Reference		Reference	
< $2684	2.018 (1.722–2.365)	< 0.001	1.224 (1.013–1.479)	0.036	3.147 (2.317–4.276)	< 0.001	1.753 (1.243–2.474)	0.001
Unknown	1.899 (1.461–2.649)	< 0.001	1.241 (0.911–1.690)	0.171	2.416 (1.432–4.077)	0.001	1.445 (0.812–2.571)	0.211
Job, n (%)								
Office-worker	Reference		Reference		Reference			
Others	1.878 (1.654–2.132)	< 0.001	1.203 (1.034–1.400)	0.017	2.562 (1.919–3.421)	< 0.001		
Unknown	2.077 (1.548–2.786)	< 0.001	1.241 (0.911–1.690)	0.171	3.769 (2.224–6.388)	< 0.001		
Alcohol consumption, n (%)								
0 g/week	Reference				Reference			
> 0 and < 100 g/week	0.919 (0.775–1.090)	0.333			0.830 (0.557–1.238)	0.362		
≥ 100 g/week	0.960 (0.833–1.107)	0.577			0.883 (0.636–1.225)	0.456		
Unknown	0.975 (0.637–1.494)	0.909			1.987 (.0958–4.118)	0.065		
MET, ln (continuous)	0.992 (.973–1.012)	0.425			0.940 (0.901–0.981)	0.004		
Central obesity	0.949 (0.839–1.074)	0.405			1.054 (0.794–1.400)	0.716		
Hypertension	0.977 (0.865–1.104)	0.713			1.053 (0.793–1.400)	0.720		
Diabetes	1.134 (1.004–1.282)	0.043			1.300 (0.984–1.718)	0.065		
Triglyceridemia	1.131 (1.003–1.276)	0.044			1.007 (0.761–1.333)	0.961		
Decreased HDL	1.330 (1.173–1.508)	< 0.001	1.183 (1.038–1.348)	0.012	1.074 (0.801–1.441)	0.632

**Table 4:** Variable associated with I-PSS total, voiding, and storage score.

	IPSS total score		Voiding sub-score		Storage sub-score	
	B (95% CI)	*p*	B (95% CI)	*p*	B (95% CI)	*p*
Age, years (continuous)	0.141 (0.121–0.162)	< 0.001	0.098 (0.083–0.113)	< 0.001	0.048 (0.040–0.057)	< 0.001
Amount of smoking, PY (continuous)	0.037 (0.025–0.050)	< 0.001	0.031 (0.022–0.040)	< 0.001	0.007 (0.001–0.012)	0.013
Marriage (yes vs. others)					0.287 (0.026–0.548)	0.031
Low income (yes vs. others)	0.915 (0.317–1.512)	0.003			0.454 (0.199–0.710)	< 0.001
Hypertension (yes vs. others)			− 0.306 (− 0.551 to 0.061)	0.014		

## Discussion

QoL is becoming a more important health issue and, in elderly men, worsened BPH-induced LUTS exacerbates QoL. Moreover, medical costs for relieving LUTS tend to be significantly increased because of its impact on QoL. However, because of the close relationship between aging and BPH/LUTS, no clear strategies for preventing the development of LUTS have been developed thus far, and prevention strategies for LUTS in men are urgently needed, as mentioned previously. In the current study, we aimed to demonstrate the impact of LUTS on QoL; based on this, we thoroughly assessed the impacts of MetS and lifestyle-related factors on LUTS to suggest the effective prevention strategies for LUTS in men to improve QoL.

Based on the current study, not only severe LUTS but also moderate LUTS affect sleep quality and stress in daily life. Moreover, the presence of LUTS was the most powerful variable associated with both sleep disturbance and stress, followed by younger age and marital status. In other words, preventing the development of moderate to severe LUTS, in addition to alleviating LUTS, could be important medical issues for improving mental health and daily QoL in men. In addition, urologists need to have more interest in mental health and daily QoL, because LUTS is one of the most important factors affecting sleep disorders and stress.

In this study, the amount of life-long smoking was significantly associated with the presence of moderate and severe LUTS, which is consistent with previous studies^15^. A previous study reported that nicotine might worsen LUTS by reducing bladder flow and urothelial hypoxia^15^, which supports the results of the current study. Interestingly, the impact of the amount of life-long smoking, not current smoking status^16^, on voiding symptoms was also observed in the current study, which supports the need for early education on the adverse effects of smoking on LUTS, although these remain to be validated in future studies. Because only a few previous studies have assessed the relationship between smoking and LUTS and these studies generally showed inconsistent results, the findings of the current study could be help clinicians and patients based on a detailed analysis of various factors related to LUTS.

In addition to age, several socioeconomic statuses showed a close relationship with the presence of moderate to severe LUTS in this study, which is similar to previous studies^16,17^. This might be due to the relationship between psychological stability and LUTS. In a previous studies satisfaction with life and presence of depression was reported to be associated with LUTS progression^18,19^, which supports these results. In the current study, we revealed that socioeconomic status, including marital status and income level was significantly associated with storage symptom, but not with voiding symptom. These findings thought to come from the association between storage symptom and mental health as previously reported^20^. In addition because widowhood reported to be associated with a greater likelihood of progression of LUTS^21^, clinicians should carefully evaluate the LUTS severity and its progression, especially storage symptoms, while they treat patients who lost their spouse.

Based on the current study, alcohol consumption and exercise were not associated with the presence of moderate to severe LUTS in men aged ≥ 40 years. In other words, the prevention of moderate to severe LUTS seemed to be achieved by life-long lifestyle modification and by maintaining a stable socioeconomic status, not by short-term daily life modification. However, because alcohol consumption and exercise were determined as factors associated with decreased sleep disorder, the importance of appropriate alcohol consumption restriction and daily exercise to improve QoL should not be overlooked, especially in mid-aged men. Among MetS and metabolic components, decreased HDL cholesterol was the only variable related to moderate to severe LUTS, which agrees with a previous study^9^. Interestingly, the presence of hypertension was negatively associated with voiding symptom severity, but not with storage symptoms. These findings might be due to the effects of alleviating LUTS by angiotensin II receptor blocker medication for hypertension as described in previous studies^22^ although these need to be verified in future studies.

The current study was limited by its cross-sectional study design, although a large number of men who underwent routine health check-ups were included in the analysis. In addition, because of the study design, we could only suggest the relationship between lifestyle and MetS-related variables with the presence of moderate to severe LUTS, and the causality needs to be verified in future studies. However, to our knowledge, this is the first large study to thoroughly assess the relationship not only between LUTS and lifestyle and MetS-related variables, but also its impact on sleep disorders and moderate to severe stress in daily life. Therefore, this study could be useful for clinicians when counselling men, who are not only worried about or experiencing moderate to severe LUTS, but are also having sleep disorders and moderate to severe stress.

In conclusions, in men aged ≥ 40 years, stable socioeconomic status, including married marital status, office worker, and high income, are negatively associated with the presence of moderate to severe LUTS, especially storage symptom. In addition, quitting smoking might be effective lifestyle modification strategies for preventing moderate to severe LUTS and eventually improving quality of life.

## Methods

### Study population

From 2014 to 2020, men who underwent health check-ups at our institute were eligible for the current study. Among these, 10,353 men who completed the I-PSS questionnaire were initially selected for the study. Then, 3477 duplicated cases were excluded from the analysis. In addition, 1263 men aged < 40 years and 165 men who did not undergo body measurements were excluded. After excluding 54 men who were taking medications for BPH, 40 men with cerebrovascular disease, and 26 men with chronic kidney disease, 5,355 men were finally included in the analysis. The current study was approved by the institutional review board of the Boramae Medical Center and the informed consent has been waived by the institutional review board of the Boramae Medical Center. In addition, all methods were performed in accordance with the relevant guidelines and regulations.

### Health check-up

Men who underwent health check-ups at our institute routinely performed the Korean version of I-PSS^23^, Pittsburgh sleep quality index (PSQI) questionnaires^24^, and modified Korean-translated brief encounter psychosocial instrument (BEPSI-K)^25^. Demographic characteristics, including age, body weight, and height, were measured, and social history and lifestyle-related factors, such as smoking, drinking, marital status, job, and income, were collected before medical check-up. We divided the job into three groups (office worker vs. others vs. unknown), marital status into three groups (yes vs. no (including single, divorced, and separated) vs. unknown), and monthly income into three groups (≥ $2684 vs. < $2684 vs. unknown). In addition, smoking status was divided into two groups (current smoker or not), while drinking status was divided into four categories according to weekly alcohol consumption (0 g vs. < 100 g vs. ≥ 100 g vs. unknown). The metabolic equivalent of task (MET) was calculated and log-transformed due to its deviated distribution. Using the NCEP ATP III definition, MetS was defined as the presence of any of the three components among the five metabolic components^26^. Using PSQI questionnaires, we determined the presence of sleep disorders as ≥ 5 scores^27^. Using BEPSI-K questionnaires^28^, the presence of moderate to severe stress was defined as a mean score of ≥ 1.8^29^. Among 5,355 men, 827 patients underwent transrectal ultrasound to measure prostate volume, and the impacts of metabolic components and lifestyle-related variables on prostate volume were analyzed using these men.

### Statistical analysis

We divided the participants into three groups according to their I-PSS score (1–7: none to mild vs. 8–19: moderate vs. 20–35: severe). Characteristics are presented as mean ± SD for continuous variables and frequency with proportion for categorical variables. Continuous and categorical variables were compared using the ANOVA test and chi-square test, respectively. We performed univariate and multivariate analyses to reveal the impact of LUTS on sleep disorders and stress. Because both moderate and severe LUTS showed significant impacts on worsening sleep quality and stress status, we determined the variables associated with the presence of moderate to severe LUTS. Patients were subdivided according to age groups, and the proportion of men with moderate to severe LUTS according to lifestyle-related variables, MetS, and metabolic components were calculated and compared. Univariate and multivariate logistic regression analyses were performed to determine the variables associated with the presence of moderate-to-severe LUTS. Variables with *p* < 0.2 in the univariate analysis were included for multivariable analysis using backward elimination. Because unknown categories in marital status, income, and job showed similar odds ratios compared with others, < $2,684, and others, respectively, marital status, income, and job were re-categorized into two groups for the linear regression analysis. The variables associated with total I-PSS, voiding, and storage sub-scores were determined using univariate and multivariate linear regression analyses. In this study, *p* < 0.05 was considered statistically significant. All statistical analyses were performed using IBM SPSS Statistics version 26.
